# The immunology of diabetic cardiomyopathy

**DOI:** 10.3389/fendo.2025.1542208

**Published:** 2025-04-07

**Authors:** Ming Song, Honggang Dai, Quan Zhou, Xiao Meng

**Affiliations:** State Key Laboratory for Innovation and Transformation of Luobing Theory, Key Laboratory of Cardiovascular Remodeling and Function Research of MOE, NHC, CAMS and Shandong Province, Department of Cardiology, Qilu Hospital of Shandong University, Jinan, China

**Keywords:** diabetes, diabetic cardiomyopathy, immunity, cardiac fibrosis, immune cells

## Abstract

Diabetic cardiomyopathy is a notable microvascular complication of diabetes, characterized primarily by myocardial fibrosis and functional abnormalities. Long-term hyperglycemia induces excessive activation and recruitment of immune cells and triggers the cascade of inflammatory responses, resulting in systemic and local cardiac inflammation. Emerging evidence highlights the significant roles of immunology in modulating the pathology of diabetic cardiomyopathy. As the primary effectors of inflammatory reactions, immune cells are consistently present in cardiac tissue and can be recruited under pathological hyperglycemia circumstances. A disproportionate favor to proinflammatory types of immune cells and the increased proinflammatory cytokine levels mediate fibroblast proliferation, phenotypic transformation, and collagen synthesis and ultimately rise to cardiac fibrosis and hypertrophy. Meanwhile, the severity of cardiac fibrosis is also strongly associated with the diverse phenotypes and phenotypic alterations of the immune cells, including macrophages, dendritic cells, mast cells, neutrophils, and natural killer cells in innate immunity and CD4^+^ T lymphocytes, CD8^+^ T lymphocytes, and B lymphocytes in adaptive immunity. In this review, we synthesized the current analysis of the critical role played by the immune system and its components in the progression of diabetic cardiomyopathy. Finally, we highlight preclinical and clinical immune targeting strategies and translational implications.

## 1 Introduction

Diabetes mellitus (DM) is a metabolic disorder associated with increased morbidity and mortality rates over the past several decades. The International Diabetes Federation (IDF) reported that 451 million adults developed DM in 2017 around the world, which is projected to reach 693 million by 2045 (1). Over 90% of cases are type 2 DM (T2DM), which is characterized by hyperglycemia and insulin resistance (2). T2DM has been a global health concern with the potential to cause a wide range of clinical complications affecting multiple organs, including the heart, kidneys, and retinopathy. These complications can lead to disability and death in some cases. Epidemiological studies have indicated that the most significant challenge in T2DM is cardiovascular complications, which account for more than half of deaths in diabetic patients (3).

Diabetic patients are more susceptible to cardiac structural and functional abnormalities, collectively known as diabetic cardiomyopathy (DCM). DCM is a notable microvascular complication of DM. It is characterized by extensive cardiac fibrosis, left ventricular hypertrophy, cardiomyocyte apoptosis, and microangiopathy, which may occur independently of other recognized cardiac risk factors, such as coronary artery disease and hypertension (4). DM and heart failure are frequent concomitant conditions, each of which independently increases the risk of the other (5). It has been documented that individuals with DM have a two- to four-fold increased risk of developing heart failure compared to the general population (5). Moreover, a 1% increase in glycated hemoglobin A1c levels elevates the risk of heart failure by 8% in patients with T2DM (6). It is noteworthy that heart failure is correlated with the presence of DCM in diabetic patients and that DCM further exacerbates cardiac dysfunction and increases the risk of heart failure (7). DCM has emerged as the leading cause of death among patients with T2DM (8).

There has been a notable increase in the attention paid to DCM owing to its rapidly increasing prevalence. The pathological mechanisms underlying DCM are intricate and complex. Accumulating evidence indicated that several interconnected mechanisms, including endothelial dysfunction, oxidative stress, inflammation, cardiomyocyte apoptosis, and mitochondrial dysfunction, are involved in the onset and development of DCM (9). Despite considerable research, the precise pathogenesis of DCM remains to be elucidated. Furthermore, currently available therapies for DCM remain limited, primarily because of the incomplete understanding of disease progression. Given these considerations, a better understanding of the potential pathogenesis of DCM and optimal management of this disease is highly warranted.

The immune system develops in humans at three weeks of gestation, maintaining tissue integrity and repair and preserving the body’s overall homeostasis (10). Recent studies indicated that the alterations in the immune system function are implicated in the pathogenesis of T2DM and DCM (11). In T2DM patients, hyperglycemia adversely affects immune cells, which may disturb their phenotype and function (12). Immune dysfunction is prevalent in patients with DCM, who consistently exhibit altered numbers and functions of immune cells (13). Currently, the field of immunology has been the subject of considerable research in DCM. A detailed understanding of the autoimmune mechanisms that underpin DCM may facilitate the advancement of medical progress and the formulation of treatment recommendations for this disorder. This study aimed to discuss recent advances in understanding the immune pathogenesis of DCM. Furthermore, we examined the current status and potential avenues of immune-targeted therapies.

## 2 Immune cells in the heart

The heart contains a multitude of cell populations, including endothelial cells (approximately 45% of the total cell count), cardiomyocytes (30%), fibroblasts (11%), smooth muscle cells, pericytes, and mesenchymal cells (8%) as well as a plethora of immune cells (6%) that maintain cardiac homeostasis and function (14). Each cell population was responsible for a specific function. Of particular significance, various cell types within the heart engage in intercellular communication while simultaneously maintaining cardiac homeostasis.

Immune cell subpopulations, including macrophages, dendritic cells (DCs), mast cells, natural killer (NK) cells, and lymphocytes, are consistently present in cardiac tissue. Conversely, neutrophils and monocytes are absent in healthy cardiac tissue but can be recruited to damaged myocardium (14). Available evidence has demonstrated that immune cells present within the myocardium exert actions to maintain cardiac development and function (15). In addition, immune cells can directly affect other resident cells, including cardiomyocytes, vascular endothelial cells, and fibroblasts. This intercellular communication between immune cells and other cell types is essential for maintaining structural integrity and physiological homeostasis of the heart (16). However, the precise nature of the communication between cell types remains unclear.

Hyperglycemia in diabetes is thought to cause dysfunction of the immune cells. In macrophages, high extracellular glucose promoted proinflammatory gene expression through glycolysis-dependent mechanisms (17). High glucose activated transforming growth factor-β (TGF-β) and promoted T helper-17 cell differentiation by improving mitochondrial ROS (18). The roles of metabolites of glycolytic metabolism, such as citrate, succinate, and itaconate, in the process of immune cell activation have been highlighted. The mechanism includes post-translational modification of proteins. Citrate favors the production of proinflammatory cytokines by macrophage and activation of DCs and NK cells. Itaconate increased the expression of engulfment mediators and enhanced the engulfment of macrophages. Succinate can bind to its receptor SUCNR1 to sustain the proinflammatory phenotype of macrophage. Moreover, succinate-SUCNR1 signaling activated DC2 to support their antigen-presenting capacity. Activated immune cells prefer to use lactate to support their activation. Lactate shuttles across cytoplasmic and intracellular compartments to accomplish its functions. The accumulated lactate in the microenvironment acts as a signaling molecule mediating immune cell activation. Macrophages rely heavily on glycolysis to perform their functions, which was further confirmed by decreased inflammatory cytokine release and migration after LDHA and MCT-4 knockdown (19). Lactate was demonstrated as a new epigenetic regulator via histone lactylation (20). Histone lysine residue lactylation can be stimulated by glycolysis to further activation of macrophages. In ex vivo T cell activation assays, lactate increased the secretion of cytokines, such as IFN-γ, IL-2, and TNF-α (21).

## 3 The crosstalk of the inflammation and immunity in DCM

T2DM is a chronic, low-grade inflammatory disease (22). Systemic and local cardiac inflammation induced by hyperglycemia can exacerbate insulin resistance and loss of beta cell function, an independent risk factor for DCM progression (23). Clinical and experimental studies have established an association between the severity and prognosis of DCM and inflammation levels, emphasizing that myocardial inflammation is a causative factor in the initiation and progression of DCM (24).

The immune system protects the host against infection and maintains homeostasis. The immune system is a complex network that involves initiating, progressing, and resolving inflammatory responses following organ damage (14). Cardiac metabolic disorders and immune dysregulation have been associated with T2DM and DCM. Long-term hyperglycemia induces the excessive recruitment of innate and adaptive immune cells at lesion sites, including macrophages, mast cells, neutrophils, and lymphocytes (13). The recruitment of excessive immunocompetent cells into the myocardium triggered a cascade of cardiac inflammatory responses, which impaired the heart and promoted cardiac dysfunction (25). In all stages of DCM, immune cell infiltration into the myocardium is a commonly observed phenomenon that has been implicated in the pathophysiology of DCM (7). Cardiac inflammation can cause persistent cardiac injury, which is considered a cardinal feature of DCM (26).

Inflammation, immune response, and metabolism are highly integrated (27). Uncontrolled inflammatory stimuli can cause the accumulation of immune cells and persistent activation of the immune system. Immune cells are the primary effectors of inflammatory reactions. The accumulation and overactivation of these immune cells promote the production of proinflammatory cytokines, (such as tumor necrosis factor-α (TNF-α), interleukin-1β (IL-1β), and IL-6 driving cardiac inflammation and myocardial damage (14). A number of studies have demonstrated increased proinflammatory cytokines levels, including TNF-α, IL-1β, IL-6, macrophage chemotactic protein-1 (MCP-1), and C-reactive protein (CRP), in the cardiac tissues of DCM animal models compared to non-diabetic control animals, which was associated with cardiac fibrosis and hypertrophy (28). Thus, modulation of the inflammatory network may be an efficacious treatment for cardiac injuries caused by diabetes and a means of preventing the development of DCM. Animal studies have demonstrated that inhibition of intramyocardial inflammation can effectively attenuate myocardial interstitial fibrosis and improve cardiac function. This has led to recognizing this approach as an effective therapeutic strategy for DCM (29). Proinflammatory cytokines are released and persist, further triggering the activation of both innate and adaptive immune responses, which promote the recruitment and infiltration of immune cells in the myocardium tissues (12). Excessive infiltration of immune cells and elevated cardiac inflammation are attached together and implicated in DCM development (30).

## 4 The crosstalk of cardiac fibrosis and immunity in DCM

Cardiac fibrosis is not a distinctive disease entity; instead, it is a common pathological abnormality associated with several cardiac diseases, including heart failure, DCM, and hypertension (31). Cardiac fibrosis has been demonstrated to reduce tissue stiffness, adverse cardiac remodeling, and progression to heart failure. Fibroblasts are the primary drivers of cardiac fibrosis and constitute one in about five of the non-myocardial cells in the heart. Hyperglycemia implicates the development of cardiac fibrosis in individuals with DM. This process involves the proliferation of cardiac fibroblasts, conversion of fibroblasts into myofibroblasts, secretion of fibrogenic mediators, synthesis of collagen, and remodeling of the cardiac fibroblast population (32). Some animal models of diabetes have been observed to exhibit increased cardiac fibrosis before the onset of hyperglycemia (33). In patients with DM, the degree of cardiac fibrosis is primarily determined by the duration and severity of metabolic dysregulation with or without accompanying diseases (34). Cardiac fibrosis contributes to an increase in ventricular stiffness, which is associated with an elevated risk of hospitalization owing to heart failure and mortality (34). Furthermore, experimental and clinical evidence indicates that diabetic fibrotic remodeling of the heart may promote arrhythmogenesis owing to disrupted cardiac conduction and enhanced arrhythmogenicity (34). Myocardial fibrosis is primarily caused by collagen accumulation. The main collagen components in the heart are collagens I and III, which determine myocardial stiffness and compliance. Overproduction of collagen I and III is implicated in myocardial collagen network remodeling and may represent the primary pathological change in myocardial fibrosis induced by DM (35). An increase in cardiac fibrosis with notable accumulation of collagen in the heart is a prominent histopathological feature of DCM that correlates with cardiac remodeling and dysfunction (36). Cardiac fibrosis and subsequent cardiac remodeling are pivotal indicators of DCM and promote cardiac dysfunction and heart failure (37). In diabetic mice induced by STZ injection for five consecutive days, Masson’s trichrome staining demonstrated a notable increase in collagen accumulation within the cardiac perivascular and interstitial spaces when compared with control mice. Elevated protein expression of collagen I and III in the diabetic heart was also found, indicating an exaggeration of cardiac fibrosis (38). In addition, db/db mice at 20 weeks of age also exhibit cardiac fibrosis, accompanied by cardiomyocyte hypertrophy (39). Cardiac fibrosis has adverse prognostic implications in patients with DM, and inhibition of cardiac fibrosis has been demonstrated to effectively protect against DCM progression and heart failure (37). Nevertheless, despite documentation of the fibrogenic effects induced by hyperglycemia, the mechanisms underlying the development of fibrosis remain unclear.

**Figure 1:**
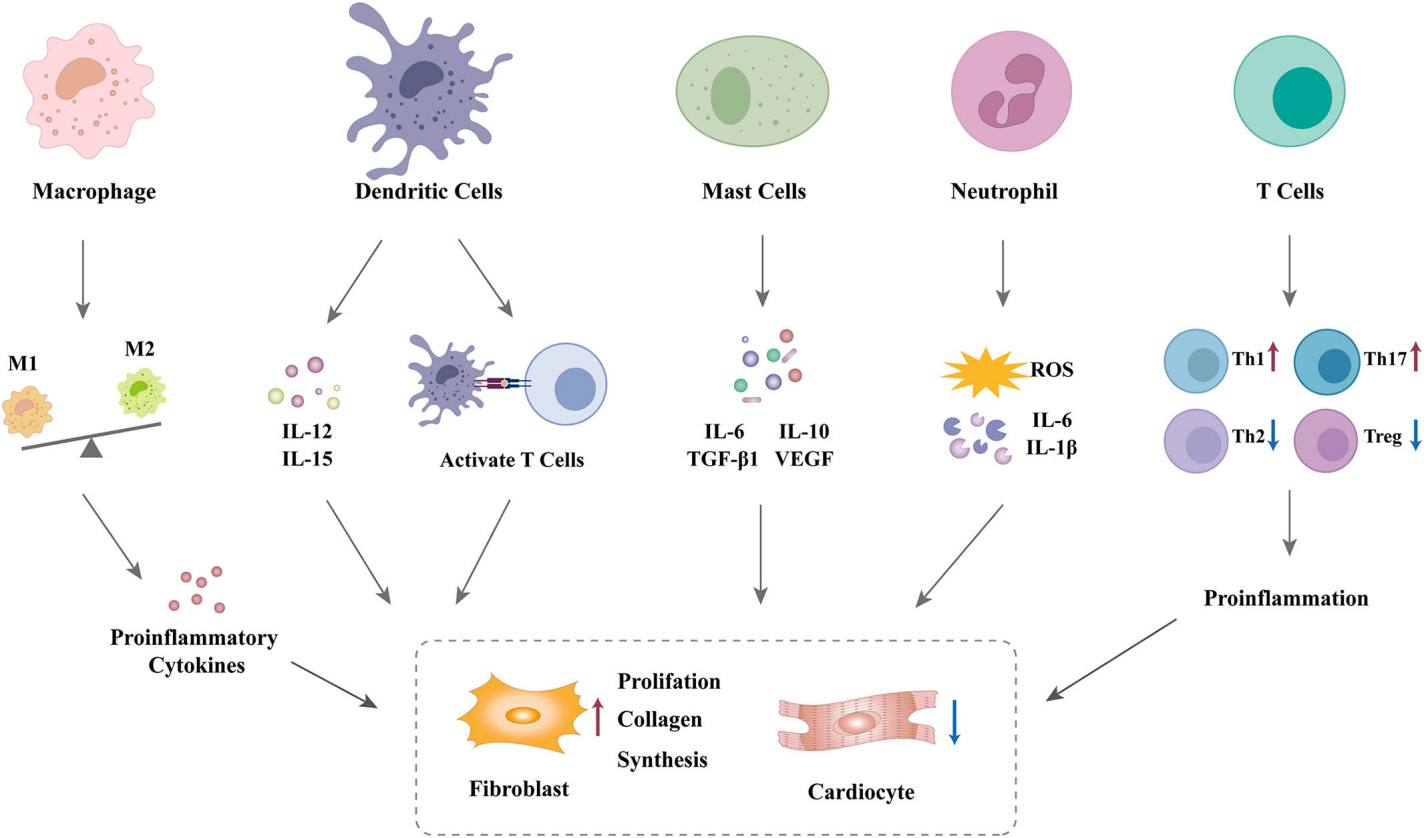
Mechanisms linking immune cells and cardiac fibrosis in DCM.

Although cardiac fibroblasts are the primary effector cells involved in cardiac fibrosis in the diabetic heart, various immune cells are also involved in the fibrogenic response. Numerous immune cells trigger fibroblast activation and promote the differentiation of fibroblasts into myofibroblasts via the secretion of a diverse range of inflammatory and profibrotic mediators (34). These mediators are regarded as significant contributing factors in the development of cardiac fibrosis (40). Therefore, it can be reasonably deduced that disruption in the communication between immune cells and fibroblasts may contribute to myocardial fibrosis in patients with DCM, as shown in **Figure 1**. Nevertheless, crosstalk between immune cells and fibroblasts in the diabetic heart remains an area of significant research interest.

## 5 Innate immunity in DCM

Innate immunity represents the initial barrier to defense against pathogen aggression and maintains tissue homeostasis (32). Innate immune cells, including macrophages, DCs, mast cells, neutrophils, and NK cells, have been implicated in the pathogenesis of DM. In hyperglycemia, innate immunity is activated by tissue injury, which impairs insulin secretion and action and promotes inflammatory responses (41). However, the role of innate immunity in DCM development remains unclear.

### 5.1 Macrophages

Macrophages are primary immune cells commonly associated with protection against infection and act as mediators of tissue damage. They can remove apoptotic and necrotic cells, maintain homeostasis, and exert an irreplaceable role in human health (42). Macrophage-mediated efferocytosis is a fundamental mechanism for the clearance of dead and apoptotic cardiomyocytes. Macrophages are heterogeneous and exhibit different phenotypes and functions depending on the surrounding microenvironment, including proinflammatory M1 and anti-inflammatory M2 states (43). M1 subsets secrete copious amounts of proinflammatory cytokines (such as TNF-α, IL-1β, and IL-6) that induce local inflammation and insulin resistance. In contrast, M2 macrophages predominantly release IL-4, IL-10, and IL-13, which can effectively counteract the actions of M1 macrophages, suppressing inflammatory responses. The equilibrium between M1 and M2 subsets is crucial in maintaining inflammatory homeostasis (44). In addition, macrophages also were divided into two categories: monocyte-derived and tissue-resident macrophages. These two groups exhibit distinct functional characteristics owing to their different cellular origins (45).

Macrophages are present within the heart, are distributed throughout the interstitial space, and are in proximity to endothelial cells (14). The resident cardiac macrophage subset has a primary origin in embryonic development (46). Cardiac macrophages are key pleiotropic cells of the innate immune system, comprising approximately half of the immune cells in the heart (47). Macrophages are determinants for tissue repair after cardiac injury. They can scavenge pathogens, promoting neovascularization in damaged cardiac tissues and eliminating apoptotic cells and damaged organelles (48).

However, persistent hyperglycemia has been observed to trigger the accumulation of macrophages in the heart, which mainly originates from the recruitment of circulating monocytes. This may cause replacement of the resident macrophage subset (49). On the other hand, elevated glucose levels impair macrophage phagocytosis, disrupt the M1/M2 balance, and favor macrophage polarization toward the M1 phenotype. This promotes the secretion of a multitude of inflammatory cytokines, chemokines, and matrix metalloproteinases (MMPs), activating various inflammatory signaling pathways (such as nuclear factor kappa-B) and exacerbating the inflammatory network (50). Under persistent hyperglycemic conditions, macrophage-mediated cardiac inflammation plays a pivotal role in the pathogenesis of DCM, contributing to cardiomyocyte hypertrophy, myocardial fibrosis, and adverse remodeling of the extracellular matrix (43). In animal experiments, there was a significant increase in the number of M1 macrophages and a decrease in the number of M2 macrophages in the myocardium of db/db mice. This suggests that macrophages in diabetic hearts are mainly polarized to the M1 phenotype (51). Furthermore, the phagocytic capacity of macrophages is impaired, and the release of lysosomal enzymes is diminished in DM (12).

Macrophage infiltration frequently occurs before the onset of cardiac dysfunction, which contributes to adverse cardiac remodeling and DCM progression. Experimental mouse models of STZ-induced DCM exhibit pathological characteristics analogous to those observed in DCM patients, including elevated glucose levels, augmented cardiac fibrosis and collagen deposition, and impaired cardiac function. These models have been used as animal models of DCM (52). In mice with STZ-induced diabetes, F4/80^+^ macrophage numbers in the heart were greater than that in non-diabetic mice, indicating that the number of cardiac inflammatory macrophages is upregulated during the course of DCM (47). In a separate rat model of STZ-induced DCM, increased infiltration of F4/80^+^ cells was also observed in the cardiac tissues. Furthermore, the number of the M1 population was significantly greater than the M2 population in cardiac tissues (30). It has been demonstrated that infiltration and activation of macrophages are involved in the development of diabetic cardiac fibrosis (34). Guo et al. demonstrated that macrophages, predominantly M1 macrophages, mediate myocardial fibrosis through IL-1β and its corresponding receptor pathway (51). In one of the *in vitro* experiments, high glucose-treated macrophages were observed to enhance the levels of fibrosis-related factors FN-1 and α-SMA in co-cultured H9C2 cells (30). Recently, Chen et al. revealed that macrophages facilitate fibrosis through phagocytosis and modulation of inflammatory responses (53). Nevertheless, the precise function of macrophages in DCM is not entirely clear.

Macrophages serve as central regulators of the immune system and communicate with other immune cells (54). Prior studies have demonstrated that macrophages can activate and proliferate lymphocytes, triggering innate and adaptive immune responses (55). Moreover, evidence indicates that macrophages engage in crosstalk with other cardiac resident cells, including cardiomyocytes, fibroblasts, and vascular endothelial cells, which may be implicated in DCM pathogenesis (54).

Resident cardiac macrophages play a non-redundant and spatially localized cardioprotective role (56). Animal studies have demonstrated that they can restrict adverse remodeling following myocardial infarction (56). Deletion of resident macrophages induces a decrease in left ventricular systolic function and adverse remodeling (56). In adults, the number of resident cardiac macrophages is always reduced because of cell apoptosis, which accounts for only a small proportion of macrophages in injured tissues. It can be reasonably deduced, therefore, that the preservation of resident cardiac macrophage subsets may be of critical importance regarding the processes of tissue repair and injury. The increased infiltration of macrophages into diabetic cardiac tissues may be attributed to the enhanced migration of peripheral macrophages, which replaces the resident macrophage population (47). However, these recruited cells cannot compensate for the depletion of resident macrophages (56). In a mouse model, the depletion of macrophages in the myocardium effectively suppressed cardiac inflammation (57). The alleviation of myocardial inflammation impedes the progression of DCM and safeguards cardiac function (44). Thus, the inhibition of macrophage infiltration in the diabetic heart may prove to be an efficacious strategy for treating DCM. In addition, Miao et al. demonstrated that M2 macrophages could safeguard co-cultured H9C2 cells from HG-induced damage, indicating that targeted M2 polarization may be advantageous for mitigating cardiomyocyte loss and myocardial injury under HG conditions (30). Furthermore, M2 macrophages have been shown to possess antifibrotic properties (58). Given the findings from these studies, macrophage-targeted therapy, which aims to preserve resident cardiac macrophages, suppress the recruitment of macrophages, and induce the transformation of macrophage polarization from M1 to M2 in the heart, may represent a promising avenue for DCM treatment.

### 5.2 DCs

DCs are antigen-presenting cells and exert a pivotal role in orchestrating immune responses (59). They secrete inflammatory cytokines (such as IL-12 and IL-15) and costimulatory molecules and activate autoreactive T cells, facilitating innate and adaptive immune responses (60). Prior studies have indicated that DCs contribute to the induction and maintenance of regulatory T (Treg) cells in peripheral blood (61).

Cardiac DCs exhibit distinct life cycles and properties. Evidence suggests that cardiac DCs partially depend on CCR2, which is distinct from the dependency observed in other tissue-resident DC subsets, such as those in the lung, liver, and kidney (62). The heart contains two subsets of conventional dendritic cells (cDCs), namely cDC1 (CD103+) and cDC2 (CD11b+). In animal studies, deletion of DC has been demonstrated to abolish CD8+ T-cell proliferation, promoting subclinical cardiac injury and overt heart failure. This process is primarily mediated by the cDC1 population (62).

A reduction in the number of DCs has been observed in patients with DM. In a study that included 15 patients with T2DM and 15 age-matched healthy persons, the frequency of DCs in the peripheral blood was measured using flow cytometry (59). The findings revealed a notable decline in the number of myeloid dendritic cell type 1 (mDC1) and plasmacytoid dendritic cells (pDC) in diabetic patients compared to healthy controls. This observation suggests that DM affects the peripheral DC pool, even in individuals with optimal glycemic control (59). Similar results were also obtained in patients with DCM. Peng and colleagues used the ImmuCellAI algorithm and observed a reduced presence of DCs in the DCM group compared with healthy control (7). Prior research has indicated that DCs serve as protective immunomodulators during the postinfarction healing process (63). It is, therefore, necessary to conduct further research to ascertain whether DCs play an immunoprotective role in DCM development.

### 5.3 Mast cells

Mast cells trigger the immune response by releasing a number of cytokines, including proinflammatory (such as IL-6 and interferon γ [IFN-γ]), anti-inflammatory (IL-10), profibrotic (transforming growth factor 1 [TGF-β1]), and antifibrotic mediators (vascular endothelial growth factor [VEGF] and prostaglandin D2 [PGD2]) (64). Like macrophages, mast cells also recruit to sites of inflammation. The recruitment of mast cells releases these mediators, which promote the accumulation of macrophages and other proinflammatory cells (52).

Mast cells are present in the heart, but their number is low. However, hyperglycemia induces the accumulation of mast cells in the heart (65). The number of mast cells is typically elevated at sites of fibrosis, enhancing their profibrotic role (64). He et al. showed that mast cells can directly activate cardiac fibroblasts and induce collagen and α-SMA expression, indicating the role of mast cells in cardiac fibrosis (52). Moreover, the involvement of mast cells in adverse cardiac remodeling has been firmly established. In animal studies, mast cell deficiency alleviated left ventricular remodeling and diastolic dysfunction (66). In mouse models of DCM induced by STZ, mice lacking mast cells exhibited a marked reduction in diastolic and systolic interventricular septal thickness and improved cardiac function, indicating that the deletion of mast cells may protect against DCM progression. Furthermore, deletion of mast cells significantly reduced cardiomyocyte apoptosis, macrophage accumulation, inflammatory cytokine production, and collagen expression in the myocardium. These findings suggest that these processes may be the primary mechanisms through which the development of DCM is suppressed (52). Interestingly, Uemura et al. showed that mast cell-deficient mice exhibited a decreased incidence of hyperglycemia-induced atrial fibrillation and interatrial conduction time as well as a diminished extent of atrial fibrosis, macrophage infiltration, and mRNA levels of TNF-α, MCP-1, IL-1β, TGF-β, and collagen-1 in the left atrium in STZ-induced DCM (65). These findings underscore the involvement of mast cells in hyperglycemia-induced atrial fibrillation, which may be attributed to amplified cardiac inflammation and fibrosis.

However, some studies have yielded contradictory results, indicating that mast cells may also possess antifibrotic properties. Joseph et al. demonstrated that mast cell-deficient rats exhibited adverse cardiac remodeling and myocardial fibrosis induced by hyperhomocysteinemia compared with control rats, indicating that mast cells may exert a protective effect during the progression of cardiac fibrosis (67). Given these discrepancies, it can be posited that mast cells may not be inherently detrimental but possess a dual role in the context of cardiac fibrosis. Further research is needed to approve the role of mast cells.

### 5.4 Neutrophils

Neutrophils represent a crucial element of the innate immune system and serve as the primary line of defense at inflammatory sites. Although neutrophils are absent in healthy cardiac tissues, myocardial damage and inflammation can induce the activation and infiltration of neutrophils into inflamed cardiac tissue (68). Upon reaching the inflammatory site, neutrophils secrete a variety of inflammatory mediators, including cytokines and chemokines, which facilitate neutrophil migration, phagocytosis, and generation of reactive oxygen species (ROS) (69). Previous studies have demonstrated that neutrophils in diabetic patients secrete greater quantities of proinflammatory cytokines (such as TNF-α and IL-1β) and ROS than those in the healthy population (70). Excessive production of cytokines and ROS facilitates cardiac tissue injury and enhances susceptibility to invasive microorganisms (71).

The neutrophil-to-lymphocyte ratio (NLR) is a marker of inflammation frequently used in clinical research. In a cross-sectional study comprising 507 patients with T2DM, patients with DCM exhibited a higher NLR than those without cardiac dysfunction, indicating a positive correlation between NLR and DCM occurrence (72). Furthermore, the duration of T2DM was more prevalent among individuals in the upper three quartiles of NLR (72). Based on these findings, NLR may serve as an effective and accurate biomarker for predicting DCM. Nevertheless, no discernible correlation has been discerned between neutrophil count and the incidence of DCM in patients with T2DM (72).

### 5.5 NK cells

NK cells are the primary effectors of the innate immune system and participate in the host defense. A study of patients with chronic heart failure revealed a notable decrease in the number and cytotoxic capacity of circulating NK cells compared with healthy individuals. A deficiency in NK cells was associated with the severity of left ventricular dysfunction (73). Subsequent animal studies demonstrated that NK cell depletion with an anti-asialo GM1 antibody exacerbated myocarditis severity and cardiac fibrosis in BALB/c mice, suggesting that NK cells can efficiently suppress cardiac inflammation and fibrosis by limiting eosinophil infiltration (74). Hyperglycemic conditions have been shown to influence NK cell activity. NK cells isolated from mice afflicted with both T1DM and T2DM exhibited reduced cytotoxicity relative to those isolated from control mice. Furthermore, an increase in the duration of hyperglycemia resulted in a corresponding decrease in cytotoxic activity (75). A recent report by Wang et al. demonstrated that NK cell-derived exosomes protect against insulin resistance and inflammation in mice with T2DM, indicating a potential role of NK cells in the pathogenesis of T2DM (76). Although direct evidence for the role of NK cells in DCM is limited, NK cells might be potential cellular effectors in the treatment of DCM owing to their anti-inflammatory and antifibrotic properties.

## 6 Adaptive immunity and lymphocytes in DCM

**Figure 2:**
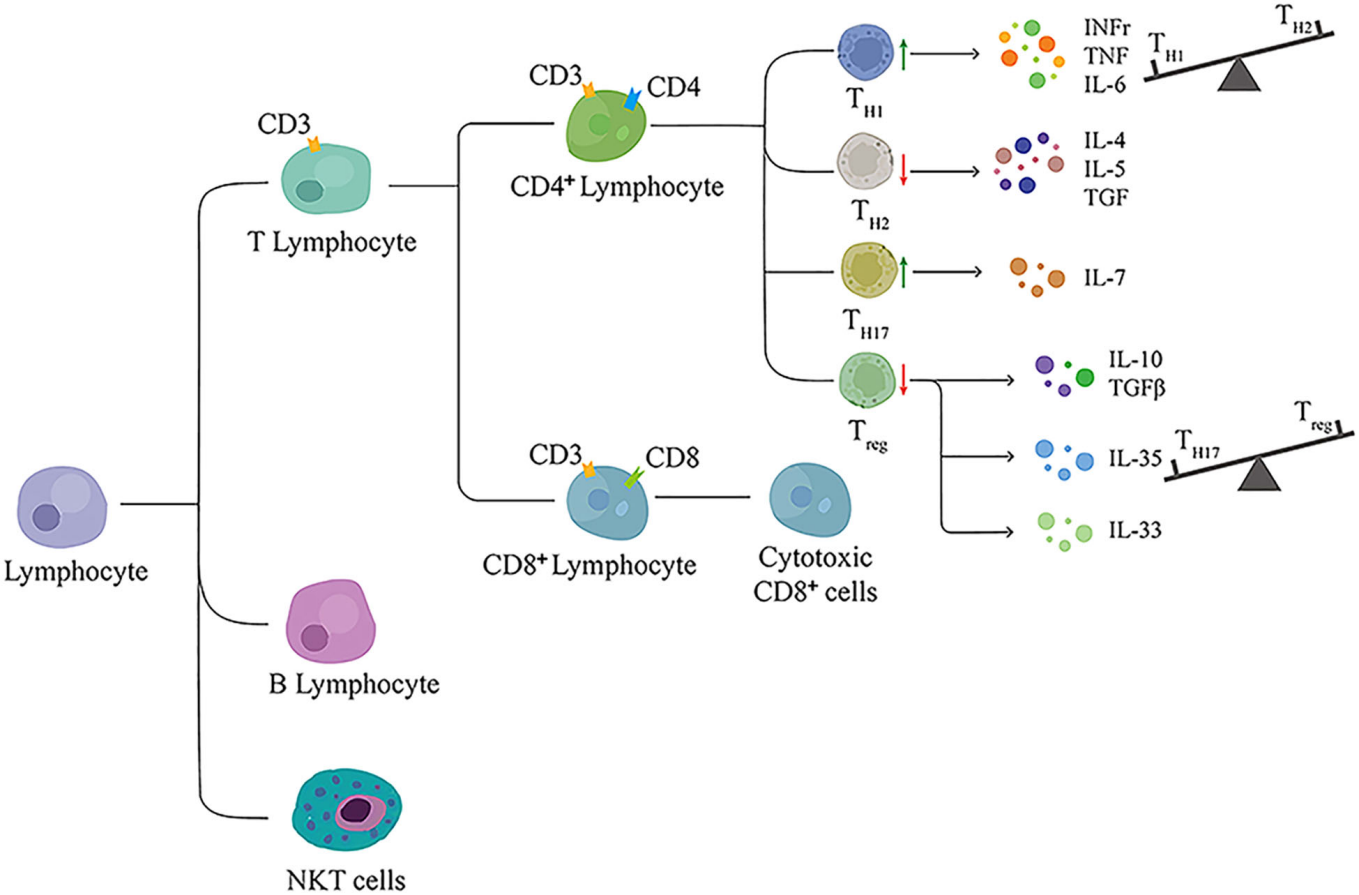
The main categories of lymphocyte contributing to DCM.

Evidence suggests that T2DM affects the equilibrium of adaptive immune cell populations, with an increase in proinflammatory cell subpopulations and a reduction in anti-inflammatory cell subpopulations (77). The adaptive immune system principally depends on lymphocytes to perform their functions. Lymphocytes constitute a heterogeneous family of adaptive immune cells that can be divided into two major categories: T and B cells. Evidence indicates that lymphocytes, both T and B cells, are implicated in cardiac fibrotic conditions and DCM progression, as shown in **Figure 2**. However, Huang et al. analyzed 570 patients with T2DM, with or without DCM, and found no association between lymphocyte count and DCM occurrence (72).

### 6.1 T lymphocyte cells

T lymphocytes play a pivotal role in the pathogenesis of insulin resistance. Deletion of T cells using a CD3-specific antibody has been demonstrated to prevent the development of insulin resistance in mice (78). In patients with T2DM, T cells skew toward proinflammatory subsets that facilitate the inflammatory response by increasing proinflammatory cytokine secretion (79). In DCM, T cells undergo aberrant alterations, and T-cell-mediated immunity is postulated to be a contributing factor to the pathogenesis of this disease (80). It has been suggested that T-cell function is a crucial modulator during the progression of DCM. Emerging evidence from clinical and animal studies indicates that T-cell infiltration in the diabetic myocardium is increased, which has been linked to an elevated risk of developing DCM (81). Furthermore, T cells stimulate the proliferation of profibrotic cells and exacerbate cardiac fibrosis through the release of fibrogenic cytokines, which contribute to DCM progression (82). On the coronary, the depletion of T cells within the circulatory system or the myocardial tissue markedly ameliorates cardiac fibrosis and preserves cardiac function in mouse models of STZ-induced DCM, indicating that mature T cells are involved in the pathophysiology of DCM (83). Therefore, it can be concluded that T-cell deletion mediates a cardioprotective role in DCM and that T-cell-based treatment strategies may protect the heart against diabetic injury.

#### 6.1.1 CD4+ T lymphocytes

T lymphocytes are subdivided into two main categories: CD4+ and CD8+ T cells. CD4+ T cells are generally classified into discrete functional subsets, including proinflammatory Th1 and Th17 cells and anti-inflammatory Th2 and Treg cells. Differentiation of T-cell subpopulations is contingent upon their distinctive physiological attributes and the production of specialized cytokines. CD4+ T cells demonstrate adaptability and phenotypic plasticity in response to varying stimuli (84). In DCM, the equilibrium between CD4+ T-cell subtypes is disrupted, resulting in a proinflammatory phenotype.

##### 6.1.1.1 Th1 cells

Th1 cells trigger cell-mediated immunity and promote the progression of islet inflammation and DM through the secretion of a multitude of proinflammatory cytokines and chemokines, including IFN-γ, TNF-α, MCP-1, IL-1β, IL-6, IL-18, and CXCL10 (85). Of particular significance, these cytokines and chemokines serve as bioactive mediators of inflammation, playing a pivotal role in physiological inflammatory responses aimed at re-establishing cardiac homeostasis. In certain pathological conditions, such as hyperglycemia, homeostasis is not restored, and the excessive production of cytokines and chemokines helps to establish a detrimental cytokine network (86). In animal studies, it was demonstrated that mice with streptozotocin-induced diabetes exhibited markedly elevated expression of interferon-gamma mRNA in cardiac tissues relative to control mice (87).

An increased Th1 response was observed in patients with DCM. Th1-driven responses have been linked to cardiac inflammation, fibrosis, and cardiac dysfunction (88). Filardi et al. indicated that Th1-driven biomolecular and functional modifications in cardiomyocytes contribute to DCM progression (89). A distinction between Th1 response and mediators in cardiomyocytes at an appropriate time may represent a potential therapeutic target for DCM progression (89).

##### 6.1.1.2 Th2 cells

As an anti-inflammatory cell subset, Th2 cells mainly secrete IL-4, IL-5, IL-10, IL-13, and transforming growth factors (TGF) to regulate antibody responses (13). Th2 cells are typically regarded as protective to avoid pancreatic islets from damage (85). *In vivo*, a balance is maintained between Th1 and Th2 subpopulations and their respective cytokines. IL-4 and IL-13 are the important an-inflammatory cytokines produced by Th2 cells. Studies have documented that IL-4 enhances insulin sensitivity and glucose tolerance (90). IL-13 may suppress leukocyte recruitment and promote M2 macrophage differentiation in the myocardium, contributing to cardiac wound healing and remodeling following myocardial infarction (91).

Nevertheless, certain animal experiments have demonstrated that IL-4 and IL-13 possess robust profibrotic characteristics, capable of activating fibroblasts, stimulating collagen synthesis, and preserving the matrix in the myocardium, which is involved in the promotion of cardiac fibrosis (92). Consequently, IL-4 and IL-13 have dual functions in cardiac remodeling. It has been postulated that a balance may exist between the anti-inflammatory and fibrogenic actions of these cytokines, which could determine the mechanism of cardiac remodeling (34).

##### 6.1.1.3 Treg cells

Treg cells are believed to use universal suppressive mechanisms to regulate innate and adaptive immune responses. These unique subpopulations control immunological tolerance and maintain immunological homeostasis via direct cell-to-cell contact and the secretion of immunosuppressive cytokines, including TGF-β, IL-10, and IL-35 (93). Treg cells can efficiently suppress the proliferation and cytokine production of T cells and modify their phenotypes for adaptation. For example, they can effectively modulate the Th1, Th17, and Th2 responses. Treg cells are found near inflamed tissues, where they can exert their suppressive function at the site of inflammation (94). Treg cells express the transcription factor Foxp3, which is a unique marker and is irreplaceable for the maturation, development, and function of Treg cells. Deficiency in Foxp3 results in the inability of Treg cells to perform their regulatory functions, which may predispose autoimmune disorders (95).

##### 6.1.1.4 The number of Treg cells is reduced in DCM

A primary characteristic of immunological disturbances is the downregulation of the Treg cell pool. Treg cell deficiency or dysfunction disrupts immune homeostasis, leading to many diseases, including atherosclerosis, abdominal aortic aneurysm, heart failure, and DM (96). In T2DM patients, the ratios of Treg/Th1 and Treg/Th17 cells are markedly downregulated, indicating a proinflammatory trend (97). In DCM, Treg cells are documented to be defective and exhibit a reduction in their numbers. In a high-fat diet-induced DCM mouse model using male C57BL/6J mice, an obvious reduction in the number of circulating Tregs was observed by flow cytometry (80). In accordance with the reduction in Treg cell numbers, Foxp3 mRNA expression in peripheral lymphocytes was also diminished, indicating a decline in the Treg subpopulation in DCM (80). Furthermore, the levels of Treg cell-associated cytokines (TGF-β) in the plasma were markedly reduced in DCM mice relative to normal controls (80).

A few studies were conducted to explore the mechanism of the reduction in Treg cells in DCM. Treg cells also express insulin receptors. Prior studies showed that elevated insulin levels activated the AKT/mTOR signaling pathway in Treg cells, inhibited IL-10 production, and impaired the capacity of Treg cells to suppress TNF-α secretion by macrophages, which contributes to heightened inflammatory responses (98). Recently, Han et al. revealed that insulin stimulation markedly suppressed CTLA-4 mRNA expression and Treg cell differentiation via the PI3K-Akt signaling pathway (80). Nevertheless, the precise mechanisms underlying the reduction in Treg cells in DCM remain elusive.

##### 6.1.1.5 Adoptive Treg cells prevent the development of DCM

Insulin resistance is independently associated with left ventricular diastolic dysfunction and can predict the occurrence of heart failure (99). The relationship between Treg cells and insulin resistance has garnered significant attention in recent years. Tregs have been demonstrated to improve insulin resistance. Compared to the control subjects, the number of natural Tregs was markedly reduced in obese patients with insulin resistance (100). In a diabetic mouse model, deficiency in Treg cells resulted in exaggerated insulin resistance with elevated blood glucose levels and impaired insulin sensitivity. Conversely, administration of Treg cells has been shown to improve insulin resistance (100).

Tregs have been identified in resting cardiac tissues (42). Tao et al. demonstrated that a ketogenic diet for 12 weeks promoted cardiac fibrosis in db/db mice, which was associated with the suppression of Treg cell numbers, differentiation, and function (39). Enhancing the percentage of circulating Treg cells may have a cardioprotective role in STZ-induced DCM models, which is associated with blunted cardiac inflammation and fibrosis (87). Therefore, Treg cell expansion may be beneficial in the progression of DCM.

Our recent study has contributed to a deeper understanding of the beneficial role of Treg cells in DCM. The administration of Treg cells protects against the progression of DCM in a db/db mouse model, as evidenced by the attenuation of cardiac hypertrophy, myocardial damage, and improvement of cardiac function (101). As indicated in this study, the cardioprotective role of Tregs may be attributed to decreased intramyocardial inflammation, oxidative stress, and cardiomyocyte apoptosis (101). In addition, Treg cell administration for 12 weeks in db/db mice resulted in a notable reduction in cardiac collagen deposition, as assessed by Sirius red and Masson trichrome staining. This came with a decline in the mRNA and protein expression of collagen I and III in both *in vivo* and *in vitro* experiments, suggesting that myocardial fibrosis may be attenuated following Treg cell treatment (101). Considering the central role of cardiac inflammation and fibrosis in DCM development, the protective effect of Tregs on DCM is probably achieved through their anti-inflammatory and antifibrotic properties. Given that the avoidance of cardiac dysfunction and the maintenance of myocardial protection represent pivotal objectives in the treatment of DCM, this study offers novel insights into the role of Treg cells in DCM therapies.

##### 6.1.1.6 Treg cell-associated cytokines in DCM

Treg cells secrete anti-inflammatory cytokines, including TGF-β, IL-10, and IL-35, which are essential for Treg cell-mediated immunosuppression. IL-10 possesses anti-inflammatory properties that can regulate the inflammatory responses triggered by pathogens or foreign particles. In one study by Tao et al., 20-week-old db/db mice exhibited markedly reduced serum levels of IL-10 and IL-4 compared with age-matched C57/BL mice (39). Another animal study demonstrated a significant reduction in IL-10 levels in heart lysates of STZ-induced diabetic rats (9). Therefore, targeting IL-10 in the development of DCM may be beneficial.

TGF-β plays a pivotal role in achieving maximum Treg suppression. Deficiency of TGF-β receptor II in T cells results in the early onset of diabetes in a transgenic mouse model of autoimmune diabetes, which was associated with enhanced T cell priming, increased accumulation of pancreas-infiltrating inflammatory monocytes, and elevated levels of proinflammatory cytokines (102). However, TGF-β is a profibrotic cytokine that promotes excessive production of collagen and induces myocardial fibrosis (31). Ample evidence has supported the role of TGF-β in cardiac repair and remodeling. Although TGF-β appeared to play a beneficial role in cardiac repair due to its anti-inflammatory properties, excessive production of TGF-β has been linked to adverse remodeling and cardiac dysfunction (103). TGF-β signaling cascades are activated in the diabetic heart. During DCM progression, hyperglycemia promotes cardiac fibrosis and collagen deposition via the TGF-β-dependent Smad signaling pathway (104). Previous studies have focused on TGF-β as a prospective target to treat DCM. Inhibition of the TGF-β1/Smad pathway can effectively prevent DCM development in diabetic rat models (105). In a separate animal experiment, age-matched TGF-β1-deficient mice exhibited a reduction in myocardial fibrosis and a greater incidence of complications than 24-month-old wild-type control mice. These findings suggest that TGF-β1 deficiency can effectively improve age-related myocardial fibrosis and left ventricular complications (106).

TGF-β is a principal regulator of fibroblast phenotype and function (103). However, the impact of TGF-β on cardiac fibroblast proliferation has yielded disparate outcomes in prior studies. Dobaczewski et al. demonstrated that TGF-β1 markedly inhibits murine cardiac fibroblast proliferation *in vitro*, a process mediated by Smad3 signaling (107). In contrast, Shu et al. reported that TGF-β1 induces cardiac fibroblast proliferation and migration (108). This discrepancy in findings may be attributed to the differentiation state of the fibroblast populations (31). Given the multiplicity of TGF-β on these resident cells in the heart, discerning their biological actions during the progression of DCM represents a significant challenge.

In addition, Treg cells secrete IL-35, which is required to maintain their phenotype and maximal suppressive activity (109). IL-35 has potent immunosuppressive and anti-inflammatory functions, blocks the differentiation of Th17 cells, and dampens the immune assault on β-cells (109). Recently, Saad et al. reported that patients with diabetes and heart failure exhibited reduced IL-35 mRNA expression levels in the blood compared with healthy subjects (110). In addition, a negative correlation was observed between IL-35 and HbA1C% in patients with diabetes and heart failure (110). Given that IL-35 inhibits mitochondrial ROS production and protects cardiomyocytes against apoptosis, it represents a promising therapeutic target for DCM (111).

Despite evidence derived from animal studies indicating that Treg cells may exert beneficial effects in DCM, the relatively low number of Treg cells, coupled with the potential for pan-immunosuppression, render the clinical application of Treg cells a challenging prospect (112). To date, the application of Treg cell therapy for DCM has been limited by a few animal studies. Consequently, the generation of a sufficient Treg cell population for clinical use is a significant technical challenge. Furthermore, it is imperative to ascertain the safety and efficacy of repeated Treg cell administration *in vivo* before proceeding to clinical trials.

##### 6.1.1.7 Th17 cells

Th17 cells mainly secrete IL-17, an important proinflammatory cytokine that plays a pivotal role in the progression of inflammation. In mice with STZ-induced diabetes, IL-17 levels in the serum and cardiac tissue were all elevated (113). IL-17 has been demonstrated to facilitate the development of T2DM, and inhibition of IL-17 has been identified as a promising therapeutic strategy for T2DM (114). Recent research has indicated that the Th17-mediated inflammatory response contributes to the onset of DCM, while deletion of IL-17 improves cardiac function in diabetic mouse models (115). Subsequent research has demonstrated that IL-17 is involved in the course of cardiac interstitial fibrosis during DCM progression. Qi et al. found that elevated glucose levels induced cardiac fibroblast inflammation through IL-17 production (116). They also confirmed that IL-17 deficiency markedly ameliorated cardiac fibrosis and improved cardiac contractility in a DCM mouse model (116). In another STZ-induced diabetic mice model, IL-17 deletion significantly attenuated cardiac interstitial fibrosis, as evidenced by a reduction in collagen mRNA levels and collagen deposition in cardiac tissues (117). *In vitro* experiments showed that IL-17 deficiency suppressed collagen levels induced by high glucose levels in cardiac fibroblasts (117). Consequently, IL-17 inhibition may be an attractive therapeutic target for DCM, given its involvement in cardiac fibrosis. In one recent study by Yue et al., anthocyanin treatment displays a beneficial role in improving cardiac function and suppressing cardiac inflammation and fibrosis in diabetic mice by inhibiting IL-17 inhibition (118). Interestingly, Li and colleagues reported that IL-17 also participates in cardiac electrical disorders. High-frequency electrical stimuli were administered to induce ventricular arrhythmias in the diabetic mice. Deletion of IL-17 significantly reduced the incidence of ventricular tachycardia, indicating that IL-17 inhibition may represent a promising avenue to prevent diabetes-related ventricular arrhythmias (119).

Maintaining a balance between Treg and Th17 cells is essential to maintain homeostasis and prevent autoimmune diseases. Compared to mice that received a standard diet, no dramatic difference in circulating Th17 cells was found in mice with DCM induced by a high-fat diet. However, the ratio of Th17/Treg cells is markedly increased in mice with DCM (80).

#### 6.1.2 CD8+ T lymphocytes

Autoantigen-reactive cytotoxic (CD8+) T cells are one of the effector cells in T-cell–mediated immunity and are involved in the onset and progression of DCM. Zhong et al. analyzed heart samples from patients with diabetic heart failure and observed a higher abundance of CD8+ T cells in the heart samples than in those from non-diabetic heart failure patients (120). Abdullah and colleagues investigated the effects of fingolimod treatment in STZ-induced DCM mouse models and found that treatment resulted in a reduction in the number of CD4+ and CD8+ T cells and a suppression of cardiac fibrosis (82). Recently, Brassington et al. reported that CD8+ T-cell depletion markedly alleviated cardiac fibrosis inhibited cardiac apoptosis, and enhanced ventricular relaxation in hypertensive mice (121). Although direct evidence regarding the role of CD8+ T-cell depletion in DCM is limited, given the core role of cardiac fibrosis in DCM progression, inhibition of CD8+ T-cell activation may be a promising therapeutic strategy to restrict cardiac fibrosis during DCM and protect against DCM progression.

### 6.2 B lymphocytes

As antigen-presenting cells, B lymphocytes serve as conduits between innate and adaptive immunity. B cells represent a significant leukocyte population within the heart, which are important regulators of inflammation in diabetes (122). Previous research has demonstrated that B cells contribute to the maintenance of an inflammatory response in DCM (10). In one study by Peng et al., markedly increased infiltration of B cells was observed in the DCM group (7). In comparison with control mice, B cell-deficient obese mice presented a blunted systemic inflammation and insulin resistance, accompanied by an increase in the percentage of Treg cells (123). In addition, B cell-mediated immunity may cause cardiac dysfunction (8). Nevertheless, the precise mechanisms underlying the B cell-mediated regulation of cardiac function during DCM progression remain unclear. Accordingly, further experimental and clinical studies are necessary to elucidate the role of B cells in the future.

## 7 Balance of ACE-angiotensin II/ACE2-angiotensin- (1–7) systems affect the immune system as related to DCM

The renin-angiotensin system (RAS) has pleiotropic actions that contribute to diabetic cardiomyopathy. RAS has two major axes. The over-activation of the classical RAS axis in diabetics leads to the increased production of angiotensin (Ang) II, which activates the angiotensin type 1 receptor, contributing to fibrosis and cardiac remodeling. On the contrary, the counter-regulatory axes Ang 1-7 inhibit tissue fibrosis and prevent cardiac remodeling. The ACE-2/Ang 1-7 axis has an important counterregulatory effect as a depressor arm of the RAS, exerting cardioprotective actions. Moreover, it has long become clear that RAS contributes to the modulation of inflammation.

Ang II is associated with low-grade inflammation characterized by innate and adaptive immune system dysfunction. ACE and ACE3 are differentially expressed in monocytes and macrophages (124). Ang II activates nucleotide-binding oligomerization domain-like receptors with pyrin domain3 (NLRP3) in macrophages and stimulates macrophage migration and IL-1β secretion (125). There have been advances in the interaction between the Ang II and lymphocytes. Ang II directly acts on T cell AT1R and simultaneously breaks the Th1 inflammatory response (126). Furthermore, Ang II modulates T lymphocyte response, especially of Th17 and Treg response. IL-17A is the effector cytokine of Th17 immune cells. Several studies support that IL-17A mediates the pathogenetic mechanisms of Ang II in diabetes, hypertension, and cardiovascular disease models (127, 128). Treg provided protection against Ang II-induced hypertension (129). The molecular mechanism involved in the regulation of immune response by Ang II is not completely understood. Toll-like receptors (TLR) are expressed on intrinsic immune cells and activate the NF-κB proinflammatory pathway. Ang II activates both TLR3 and TLR4 and induces cardiac hypertrophy (130). The myeloid differentiation factor2 (MD2) is the TLR4 coreceptor and is required for high glucose-induced expression of ACE and ATRs. MD2 mediated Ang II-induced cardiac inflammation by directly binding to Ang II (131).

ACE2 is a membrane-bound ecto-enzyme widely distributed in tissues, including heart and blood vessels, and also could be found in plasma in soluble form. ACE2 takes part in structural and functional regulation, and genetic deletion of the ACE2 gene exhibited decreased cardiac contractility. The lack of ACE2 augmented phosphorylation of the ERK/JNK pathway and elicited the inflammatory response, leading to neutrophil infiltration and upregulation of IFN-γ, IL-6, and MCP-1. The Ang1-7 activates the angiotensin AT2 receptor (AT2R) and acts as the protective arm of RAS. ACE2 converts Ang II directly into Ang 1-7. In type 2 diabetes patients, Ang 1-7 levels were independently and negatively correlated with left ventricular dysfunction and remodeling. Ang 1-7 plays the role of anti-inflammatory factor through polarization of macrophage toward the M2 like-phenotype. Ang 1-7 diminished macrophage infiltration, adipokine, and inflammatory factor secretion. In macrophage cell culture, Ang 1-7 was capable of preventing inflammatory response induced by lipopolysaccharide (132). Treatment with Ang 1-7 reduced NF-κB activity and prevented the expression of IL-1β, IL-6, and Nalp12, preventing the development of heart failure. Ang 1-7 reduced TGF-β expression and inhibited collagen synthesis in rat cardiac fibroblasts.

## 8 Clinical trials for cardiovascular outcomes in diabetes and prospects for immune-based therapy

DCM is frequently overlooked in the early stages because of the absence of characteristic symptoms. Developing optimal therapeutic interventions to prevent DCM progression can effectively reduce the prevalence of heart failure and mortality in diabetics. However, treatment of T2DM and DCM remains largely dependent on glucose-lowering therapies. A meta-analysis included more than 30 thousand diabetes patients and compared a more intensive glucose-lowering regimen to a standard regimen. Overall, the risk of heart failure-related events did not differ significantly between intensive glycemic control and standard treatment. Strict glucose control is insufficient to prevent DCM and heart failure in diabetics (133). New pharmacological agents, including sodium-glucose co-transporter type 3 (SGLT-2) inhibitors, dipeptidyl peptidase-4 (DPP4) inhibitors, and glucagon-like peptide-1 receptor agonist (GLP-1 RA) may have effects beyond glycemic control pertinent to cardiovascular risk reduction in diabetes (134). The pleiotropic mechanisms include positive metabolic, hemodynamic, renal, and vascular effects.

SGLT2 inhibitors have a unique glucose-lowering mechanism via inhibiting glucose reabsorption in the proximal tubule. SGLT2 inhibitors have been demonstrated to have a positive effect on reducing heart failure hospitalization. Recent landmark clinical trials demonstrated the beneficial effect of SGLT2 inhibitors on cardiovascular outcomes. In the EMPA-REG OUTCOME trial, empagliflozin decreased a significant 14% relative risk reduction for the primary composite outcome, which was driven by a 38% risk reduction in cardiovascular mortality and a 35% risk reduction of heart failure hospitalization (135). The CANVAS program demonstrated that the canagliflozin treatment resulted in a significant 14% relative risk reduction in the primary composite outcome (136). The therapeutic benefit of canagliflozin was further supported by the CREDENCE trial, which showed a 34% relative risk reduction in cardiorenal outcomes in patients with type 2 diabetes and kidney dysfunction (137). This trial also confirmed a robust attenuation in the composite risk of cardiovascular death and a significant risk reduction of heart failure hospitalization. The effect of dapagliflozin in the primary prevention of diabetes was proved by the DECLARE-TIMI trial, which proved the protective effect was due to a significant 27% risk reduction of heart failure hospitalization (138). These results were corroborated by a recent meta-analysis, demonstrating a significant 23% risk reduction of cardiovascular death or heart failure hospitalization with SGLT-2 inhibitors (138).

The cardiovascular outcome trials with DPP4 inhibitors (saxagliptin, alogliptin, sitagliptin, and linagliptin) have demonstrated non-inferiority to placebo with respect to primary three major adverse cardiovascular events comprising cardiovascular death, non-fatal myocardial infarction, and non-fatal stroke. In the EXAMINE trial, alogliptin did not affect the risk of heart failure hospitalization compared to placebo (139). The TECOS trial demonstrated that sitagliptin did not increase heart hospitalization than placebo (140). CARMELINA compared the effect of linagliptin vs. placebo on major cardiovascular events in type 2 diabetes patients and high cardiovascular risk, and there was no significant effect of linagliptin treatment on the risk of heart failure hospitalization or cardiovascular death (141). Despite a consistently neutral effect on primary composite outcome, SAVOR-TIMI 53 trial revealed a statistically significant increase of heart failure hospitalization in patients with saxagliptin treatment compared to placebo (142).

Clinical trials with specific GLP1 RA agents (liraglutide, semaglutide, and albiglutide) have shown a reduction in cardiovascular outcomes but a neutral effect on the risk of heart failure. In LEADER, liraglutide treatment led to a 13% decrease in the risk of major adverse cardiovascular events and cardiovascular mortality compared to placebo (143). In SUSTAIN-6, subcutaneous semaglutide led to 26% lower cardiovascular risk (144). In Harmony Outcomes, albiglutide led to a 22% lower risk of the primary composite outcome (145). Nevertheless, there was no significant reduction in the risk of heart failure hospitalization with GLP-1 RA treatment as reported in LEADER, SUSTAIN-6, or Harmony Outcomes trials. In the LIVE study, liraglutide had a neutral effect on left ventricular ejection fraction in patients with chronic heart failure (146). In the FIGHT trial, a higher risk of death and heart failure rehospitalization was observed with liraglutide (147).

Despite the fact that new pharmacological agents have been demonstrated to confer cardioprotective benefits, the prevalence and mortality of DCM remain high. To date, targeted therapeutic strategies for DCM remain limited. It is, therefore, imperative that further investigation and development of therapeutic strategies for DCM be pursued to change this situation. Immunotherapy has shown potential as a treatment for DCM. Given the preceding experimental findings, direct immunomodulation may prove to be an efficacious approach to the management of DCM (44). Improving immune balance may prove beneficial in maintaining cardiac function in patients with DCM. For instance, the administration of Treg cells may aid in preventing DCM progression. Immune-targeted therapies may provide new avenues for advanced DCM therapy. However, the encouraging results observed in animal models and clinical and experimental studies investigating the potential of immune-targeted therapy for DCM remain limited. Therefore, more in-depth animal studies and clinical trials are warranted. Further research must determine the optimal timing and methodology for immune interventions.

## 9 Summary and perspectives

DCM is always associated with poor prognosis. The complexity and multifactorial pathogenesis of this disease continues to present significant challenges in the diagnosis and treatment of patients in clinical practice. Despite the decline in the prevalence and mortality of T2DM-related cardiovascular disease due to improved glycemic control, the cases of DCM remain persistently elevated. Therefore, gaining insight into the underlying mechanisms of DCM and developing effective interventions to halt its progression is imperative.

Dysregulation of the immune system is implicated in the pathogenesis and progression of DCM. Targeting the balance of the immune system has emerged as a potential therapeutic strategy for managing DCM. Despite substantial progress in recent years, current investigations of immunity and DCM are insufficient. Understanding the role of immunity in DCM remains challenging owing to the intricate pathogenesis of this disease. Moreover, the veracity of prior findings is still a matter of debate. Accordingly, we hope this review will facilitate a more nuanced understanding of the role of immunity in DCM progression. Given the intricate nature of immune mechanisms, further experimental and clinical studies are required to elucidate the role of immunity in DCM and investigate the efficacy of immune-targeted therapy in this disease. Together, a more precise understanding of the association between immunity and DCM may facilitate the development of more therapeutic strategies.
